# Precision Measurement Method of Large Shaft Diameter Based on Dual Camera System

**DOI:** 10.3390/s22113982

**Published:** 2022-05-24

**Authors:** Xianyou Li, Ke Xu, Shun Wang

**Affiliations:** 1 Institute of Engineering Technology, University of Science and Technology Beijing, Beijing 100083, China; leexianyou@163.com; 2 Department of Mechanical Engineering, Baotou Vocational and Technical College, Baotou 014030, China; 3 Collaborative Innovation Center of Steel Technology, University of Science and Technology Beijing, Beijing 100083, China; d202110589@xs.ustb.edu.cn

**Keywords:** precision measurement, dual camera system, crankshaft measurement

## Abstract

At present, there is a problem that the measurement accuracy and measurement range cannot be balanced in the measurement of shaft diameter by the machine vision method. In this paper, we propose a large-scale shaft diameter precision measurement method based on a dual camera measurement system. The unified world coordinate system of the two cameras is established by analyzing the dual camera imaging model and obtaining the measurement formula. In order to verify the validity of the proposed method, two black blocks in the calibration plate with a known center distance of 100 mm were measured. The mean value was 100.001 mm and the standard deviation was 0.00039 in 10 measurements. Finally, the proposed system was applied to the diameter measurement of a complexed crankshaft. The mean $\mu_{95}$ values of CMM and the proposed method were ±1.02 μm and ±1.07 μm, respectively, indicating that the measurement accuracy of the proposed method is roughly equal to the CMM.

## 1. Introduction

In industrial applications, shafts, as indispensable parts, are the most widely used parts in various mechanical devices and various engineering fields that have strict requirements for accuracy [1]. The geometric dimensions and tolerances of the shaft have important impacts on the motion performance and service life of the machine. Therefore, it is important to measure the accuracy of axes comprehensively and efficiently online [2].

**Figure 1:**
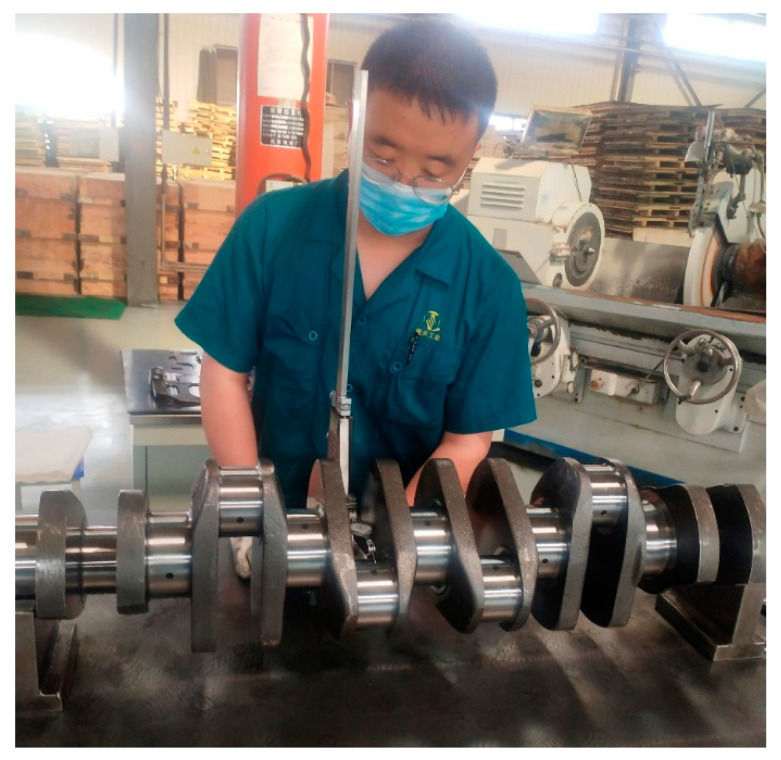
Manual measurement of crankshaft parameters.

As one of the most important parts of the engine, the crankshaft is responsible for outputting external torque. The quality of the crankshaft directly affects the performance of the car. Therefore, the measurement and error control of the crankshaft are important. The crankshaft has a complex structure and many process parameters. Among them, the shape error of the crankshaft main journal and connecting rod journal is an important indicator to measure the process quality [3,4,5,6]. Currently, the parameters of crankshaft are generally measured by manually using vernier calipers, spiral micrometers, and three-coordinate measurement [7,8,9,10], or the contact-type special measurement equipment [11,12]. The former methods need to contact the surface of the part, which will inevitably lead to a certain degree of damage to the surface of the part. The latter method has the disadvantage of expensive equipment and an inability to achieve online detection. On the other hand, personnel measurement inevitably has phenomena such as low efficiency, poor reliability, and false detection due to fatigue, and it is difficult to meet the requirements of real-time, fast, online, and non-contact detection implemented in today’s manufacturing industry. On account of the high price of three coordinate machines and special measurement equipment, manual measurement, despite its inherent flaws, is still the mainstream method at present in China. An actual manual measurement site is shown in Figure 1. a skilled worker takes 45–50 min to measure 81 parameters of a six-cylinder engine crankshaft. This high-intensity work urgently needs a new measurement technology.

Being the core technology of intelligent manufacturing, measurement technology based on machine vision has been widely used in the measurement of geometric accuracy of mechanical parts [13,14,15,16,17], as it has the characteristics of non-contact, non-destructive, high precision, and fast speed.

A variety of approaches have been developed for the precision measurement of shafts. Peng Zhen proposed a measuring method of shaft diameter based on structured light images, with a measuring diameter range of 30–50 mm and measuring accuracy of ±0.2 mm [18]. Ding Yanrong studied the key technologies of precision visual measurement of shaft workpieces, with a measuring diameter range of 11–16 mm and measuring accuracy of ±0.005 mm [19]. Yin Weiqi proposed a visual size measurement technology of shaft parts, with a measuring diameter range of 25–30 mm and measuring accuracy of ±0.06 mm [20]. Wei and Tan et al. proposed a method based on Zhang’s calibration to calibrate the internal and external parameters of the camera through the shaft with known shaft diameter; the measuring diameter range is 30–40 mm, and measuring error is less than 0.015 mm [21,22]. In the actual measurement process, the distance to be measured between the camera and the axis must be equal to the distance between the camera and the plane of the calibration board, which is difficult to guarantee. Sun et al. established a visual measurement model of shaft parts based on the principle of pinhole vision and geometric constraints, and used a plane target fixed at a specific position to calibrate the measurement system [23]. The average measurement error of the system is about 0.005 mm. By establishing an error correction model for the distance between the reference plane and the camera, the measuring diameter range is 2–10 mm, and the error is within 0.009 mm [24,25].

Based on the above works, it can be concluded that using the telecentric vision system can achieve higher-precision measurement, but the measurement range is limited. Using the pinhole vision system can achieve a large field of view, but the accuracy is limited. The algorithm needs to be optimized to improve the accuracy. In this paper, a dual telecentric camera system is proposed to measure the large shaft diameter. It has a high measuring accuracy while working in a large diameter range. This paper is organized as follows: Section 2 elaborates the measurement principle of the dual camera system, Section 3 reports the calibration results and experimental results used to test the measuring accuracy, and Section 4 provides the study’s conclusions.

## 2. Method

### 2.1. Vision Measurement Principle

**Figure 2:**
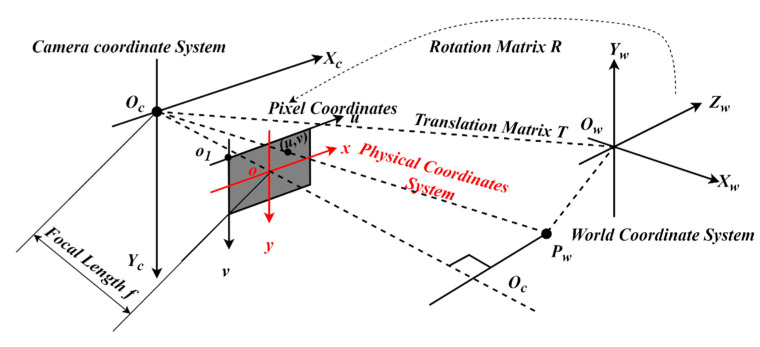
Imaging model of the pinhole camera.

The imaging model of the pinhole camera is shown in Figure 2, which shows the coordinate systems used to model the perspective projection from a 3D surface point to a 2D image point.

$O_{w}-X_{w}Y_{w}Z_{w}\;$ is the world coordinate system. The coordinate of point $P_{w}$ under the world coordinate system can be expressed as $\left(X_{w},Y_{w},Z_{w}\right)$, and its unit is mm. $O_{c}-X_{c}Y_{c}Z_{c}$ is the camera coordinate system, with the measurement unit mm. o−xy is the physical image coordinate system, the measurement unit of which is mm. $O_{1}-uv$ is the pixel image coordinate system, and its measurement unit is pixel.

Based on pinhole projection, the perspective projection from the world coordinates to the pixel image coordinate can be expressed as reported by Zhang [22] and as follows:

(1) $$S\left[\begin{array}{c} u\\ v\\ 1 \end{array}\right]=\left[\begin{array}{ccc} \alpha & \gamma & u_{0}\\ 0 & \beta & v_{0}\\ 0 & 0 & 1 \end{array}\right]\left[\begin{array}{c} R\;\;\;\;T \end{array}\right]\left[\begin{array}{c} X_{W}\\ Y_{W}\\ Z_{W}\\ 1 \end{array}\right]$$

In the equation, $\left[\begin{array}{c} \alpha \;\;\;\;\gamma \;\;\;\;u_{0}\\ 0\;\;\;\;\beta \;\;\;\;v_{0}\\ 0\;\;\;\;0\;\;\;\;1 \end{array}\right]$ represents the intrinsic camera parameters and $\left[\begin{array}{cc} R & T \end{array}\right]$ represents the extrinsic camera parameters. s is the scale factor and R,T denote the rotation matrix and translation matrix, respectively.

Assuming the measurement plane in the world coordinate system is Z=0, then the above Formula (1) can be changed to the following Formula (2).

(2) $$s\left[\begin{array}{c} u\\ v\\ 1 \end{array}\right]=\left[\begin{array}{ccc} \alpha & \gamma & u_{0}\\ 0 & \beta & v_{0}\\ 0 & 0 & 1 \end{array}\right]\left[\begin{array}{c} R\;\;\;\;T \end{array}\right]\left[\begin{array}{c} X_{W}\\ Y_{W}\\ 0\\ 1 \end{array}\right]=\left[\begin{array}{ccc} \alpha & \gamma & u_{0}\\ 0 & \beta & v_{0}\\ 0 & 0 & 1 \end{array}\right]\left[\begin{array}{c} r_{1}\;\;\;\;r_{2}\;\;\;\;T \end{array}\right]\left[\begin{array}{c} X_{W}\\ Y_{W}\\ 1 \end{array}\right]$$

In the equation, $R=\left[\begin{array}{ccc} r_{1} & r_{2} & r_{3} \end{array}\right]$. Without considering the scale factor s, it can be obtained that a point on the plane in the world coordinate system can have a one-to-one mapping relationship with the pixel point in the pixel image coordinate system. The measurement of monocular vision is based on the above principles.

### 2.2. Dual Camera Measurement System

#### 2.2.1. System Construction

Two-dimensional machine vision measurement systems can commonly be divided into two categories: telecentric vision systems and pinhole vision systems. Due to the special mechanical structure design of the telecentric lens, the image size of the object remains unchanged within the depth of field, so the measurement accuracy of the telecentric vision system usually reaches the micron level [26]. However, only the light, which is parallel to the main optical axis, can be photosensitive in the telecentric vision measurement system. Therefore, the field of view of the telecentric vision system is narrow compared to the pinhole camera.

**Figure 3:**
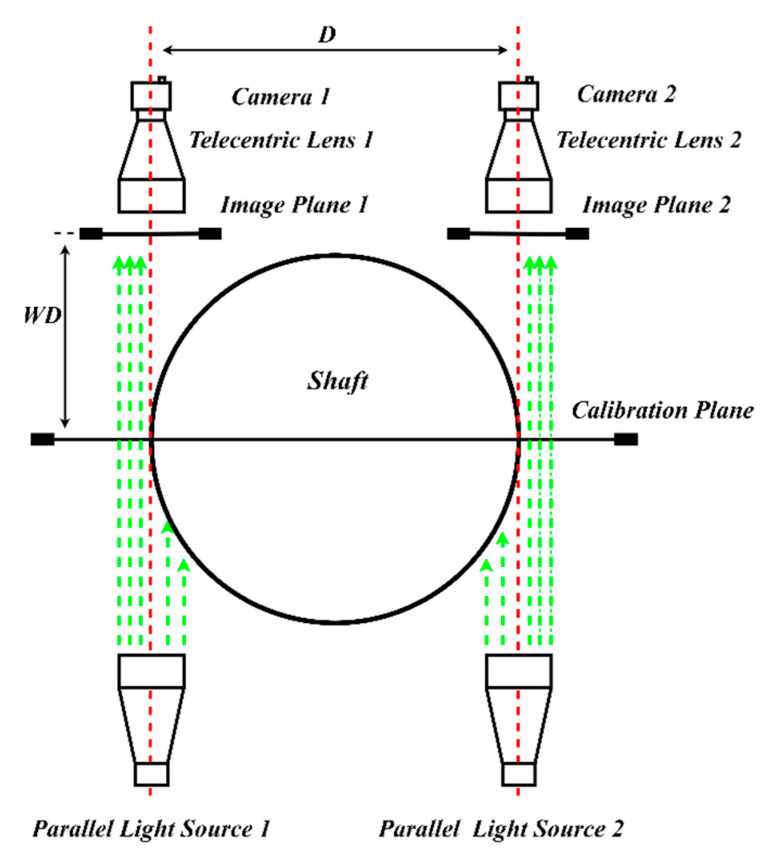
The layout diagram of the system.

The large-scale precision measurement system proposed in this paper is shown in Figure 3. This system consists of two double telecentric lenses, two high-resolution CCD cameras, and two telecentric back light sources. Assuming that the diameter of the shaft to be measured is *D*, the working distance of the object is *WD*. The two cameras are placed above the left and right edge of the shaft, and the distance between the two cameras is roughly *D* to make sure that the edge of the workpiece is in the field of view. Two telecentric back light sources are placed under the left and right edge of the shaft. The camera and lens should be selected to ensure the tolerance requirements of the measured workpiece.

#### 2.2.2. Dual Camera Measurement Principle

**Figure 4:**
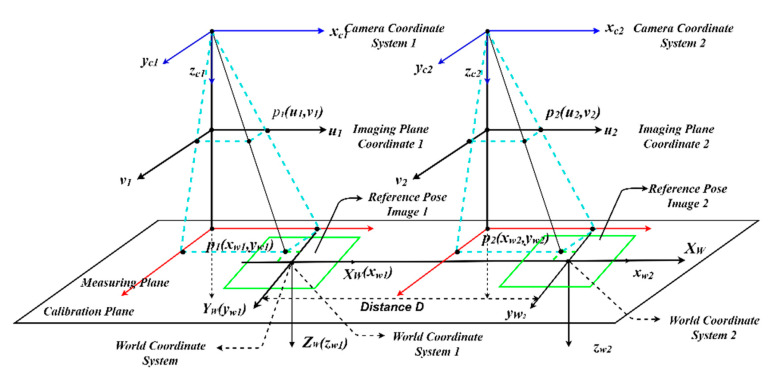
The dual camera measurement system.

Figure 4 shows the measurement principle of the dual camera system. In this paper, we use dual cameras instead of binocular vision to represent the measurement system, as the latter has two eyes capable of facing the same direction to perceive a single three-dimensional image of its surroundings. In Figure 4, the reference pose image 1 is captured by the left camera, and the reference pose image 2 is captured by the right camera. The corresponding world coordinate systems of the left and right cameras are $\left(x_{w1},y_{w1},z_{w1}\right)$ and $\left(x_{w2},y_{w2},z_{w2}\right)$, respectively. The corresponding coordinate in the world coordinate system of the pixel $P_{1}\left(u_{1},v_{1}\right)$ in the reference pose image 1 captured by the left camera is $P_{w1}\left(x_{w1},y_{w1}\right)$, while the corresponding coordinate in the world coordinate system of the pixel $P_{2}\left(u_{2},v_{2}\right)$ in the reference pose image 2 captured by the right camera is $P_{w2}\left(x_{w2},y_{w2}\right)$. We assume that there is another world coordinate system $\left(X_{W},Y_{W},Z_{W}\right)$, which is equal to $\left(x_{w1},y_{w1},z_{w1}\right)$. As the two coordinate systems $\left(x_{w1},y_{w1},z_{w1}\right)$ and $\left(x_{w2},y_{w2},z_{w2}\right)$ are on the same calibration plate, the coordinate of $P_{w1}\left(x_{w1},y_{w1}\right)$ in $\left(X_{W},Y_{W},Z_{W}\right)$ is still $P_{w1}\left(x_{w1},y_{w1}\right)$, while the coordinate of $P_{w2}\left(x_{w2},y_{w2}\right)$ in $\left(X_{W},Y_{W},Z_{W}\right)$ is $P_{w2}\left(x_{w2}+D,y_{w2}\right)$.

According to the above analysis, the pixels in the different images captured by the two cameras are unified in the world coordinate system. The corresponding distance in the world coordinate system of $P_{1}\left(u_{1},v_{1}\right)$ and $P_{2}\left(u_{2},v_{2}\right)$ is calculated by the following formula:

(3) $$S\left(p_{1},p_{2}\right)=\sqrt{{\left(x_{w1}-\left(x_{w2}+D\right)\right)}^{2}+{\left(y_{w1}-y_{w2}\right)}^{2}}$$

In the equation, $\left(x_{w1},y_{w1}\right)$ and $\left(x_{w2},y_{w2}\right)$, which is calculated by the corresponding pixel coordinates and the camera projection transformation matrix; both are coordinates in their corresponding world coordinate system.

### 2.3. Subpixel Edge Extraction Based on Interpolation

After capturing the edge image of the workpiece, the edge detection algorithm Canny operator [27] is used to obtain the edge of the image pixel. Then, according to the distribution of the grayscale of the edge neighborhood, the interpolation function is used to approximate the grayscale function (one-dimensional grayscale function) of the edge transition area to implement sub-pixel edge localization. The interpolation method is suitable for real-time online detection due to its simple operation and high positioning accuracy [28]. The Lagrange interpolation function expression is as follows:

(4) $$L\left(x\right)=\overset{n}{\underset{c=1}{\sum }}\frac{\left(x-x_{0}\right)\ldots \left(x-x_{c-1}\right)\left(x-x_{c+1}\right)\ldots \left(x-x_{n}\right)}{\left(x_{c}-x_{0}\right)\ldots \left(x_{c}-x_{c-1}\right)\left(x_{c}-x_{c+1}\right)\ldots \left(x_{c}-x_{n}\right)}f_{c}$$

In the equation, n is the total number of the interpolation points, $x_{c}$ is the interpolation point, and $f_{c}$ is the interpolation grayscale. In this paper, we use quadratic polynomial interpolation and n=3. Along the x axis of the gradient magnitude image, $x_{i}-\Delta_{x},\;x_{i},x_{i}+\Delta_{x}$ are taken as the interpolation points, and $\Delta_{x}$ is the distance. $M\left(i-\Delta_{x},j\right),M\left(i,j\right),M\left(i+\Delta_{x},j\right)$ correspond to the interpolation grayscale. Equation (4) can be written as the following function:

(5) $$\begin{array}{cc} L\left(x\right) & =\frac{\left(x-x_{i}\right)\left[x-\left(x_{i}+\Delta_{x}\right)\right]}{\left[\left(x_{i}-\Delta_{x}\right)-x_{i}\right]\left[\left(x_{i}-\Delta_{x}\right)-\left(x_{i}+\Delta_{x}\right)\right]}M\left(i-\Delta_{x},j\right)\\ & +\frac{\left[x-\left(x_{i}-\Delta_{x}\right)\right]\left[x-\left(x_{i}+\Delta_{x}\right)\right]}{\left[x_{i}-\left(x_{i}-\Delta_{x}\right)\right]\left[x_{i}-\left(x_{i}+\Delta_{x}\right)\right]}M\left(i,j\right)\\ & +\frac{\left[x_{i}-x)\right]\left[x-\left(x_{i}-\Delta_{x}\right)\right]}{\left[x_{i}+\Delta_{x})-\left(x_{i}-\Delta_{x}\right)\right]\left[\left(x_{i}+\Delta_{x}\right)-x_{i}\right]}M\left(i+\Delta_{x},j\right)\; \end{array}$$

Let dL(x)/dx=0; the *x* coordinate of subpixel edge point is:

(6) $$x_{s}=x_{i}+\frac{M\left(i-\Delta_{x},j\right)-M\left(i+\Delta_{x},j\right)}{M\left(i-\Delta_{x},j\right)-2M\left(i,j\right)-M\left(i+\Delta_{x},j\right)}.\frac{\Delta_{x}}{2}$$

**Figure 5:**
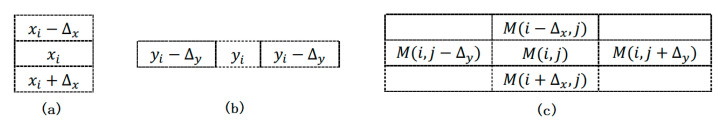
The schematic diagram of interpolation method. (**a**) The interpolation along *x* axis. (**b**) The interpolation along *y* axis. (**c**) The gray level of the interpolation point.

Similarly, along the y axis, $y_{i}-\Delta_{y},\;y_{i},\;y_{i}+\Delta_{y}$ are taken as the interpolation points, and the corresponding interpolation gray levels are $M\left(i,j-\Delta_{y}\right),M\left(i,j\right),M\left(i,j+\Delta_{y}\right)$, respectively. The schematic diagram of the interpolation method is shown in Figure 5.

The interpolation point taken along the x axis is shown in in Figure 5a, the interpolation point taken along the y axis is shown in Figure 5b, and the interpolation gray level is shown in Figure 5b.

The *y* coordinates of the subpixel edge point is:

(7) $$y_{s}=y_{i}+\frac{M\left(i,j-\Delta_{y}\right)-M\left(i,j+\Delta_{y}\right)}{M\left(i,j-\Delta_{y}\right)-2M\left(i,j\right)-M\left(i,j+\Delta_{y}\right)}.\frac{\Delta_{y}}{2}$$

The matrix form of the above equation is as follows:

(8) $$\left[\begin{array}{c} x_{s}\\ y_{s} \end{array}\right]=\left[\begin{array}{c} x_{i}\\ y_{i} \end{array}\right]+\left[\begin{array}{c} \frac{M\left(i-\Delta_{x},j\right)-M\left(i+\Delta_{x},j\right)}{M\left(i-\Delta_{x},j\right)-2M\left(i,j\right)-M\left(i+\Delta_{x},j\right)}.\frac{\Delta_{x}}{2}\\ \frac{M\left(i,j-\Delta_{y}\right)-M\left(i,j+\Delta_{y}\right)}{M\left(i,j-\Delta_{y}\right)-2M\left(i,j\right)-M\left(i,j+\Delta_{y}\right)}.\frac{\Delta_{y}}{2} \end{array}\right]$$

**Figure 6:**
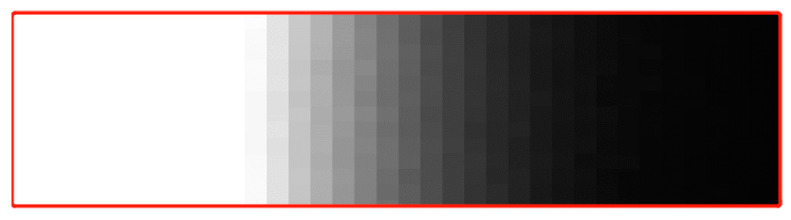
The edge transition area.

In practical application, $\Delta_{x}\;,\;\Delta_{y}$ take the particular value according to the edge transition area. In this paper, we find that the edge transition area, which is shown in Figure 6, consists of 12 pixels. Therefore, we set $\Delta_{x}=3\;\mathrm{and}\;\Delta_{y}=1$. The details of the detection results are given in Section 3.3.

## 3. Experiments and Results

In order to fully verify the effectiveness and stability of the proposed method, this section is organized as follows: Section 3.1 introduces the machine parameter requirements of crankshaft diameter, Section 3.2 builds the experimental platform, Section 3.3 calibrates the camera and the dual camera system, Section 3.4 uses the proposed method to measure the known size in the calibration board, and Section 3.5 measures the complexed crankshaft diameter by the proposed method and CMM. Finally, we analyze the measurement results.

### 3.1. Crankshaft

**Figure 7:**
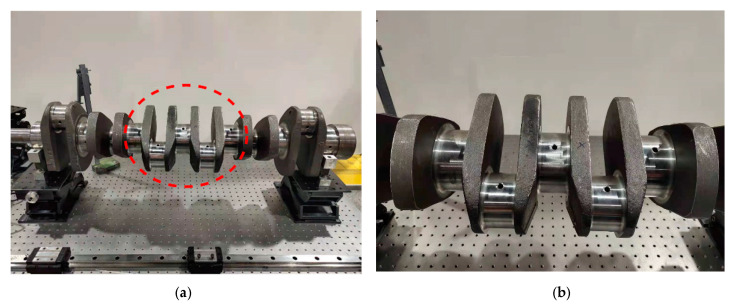
Heavy-duty truck engine 6-cylinder crankshaft. (**a**) Image of the whole crankshaft. (**b**) Image of partial crankshaft.

The measurement object in the experiment is the diameter of the main shaft journal of the heavy-duty engine by fine grinding, whose machine precision is $\phi 100_{-0.022}^{-0.01}$. The heavy-duty truck engine six-cylinder crankshaft to be measured is shown in Figure 7.

### 3.2. Experimental Platform Construction

**Figure 8:**
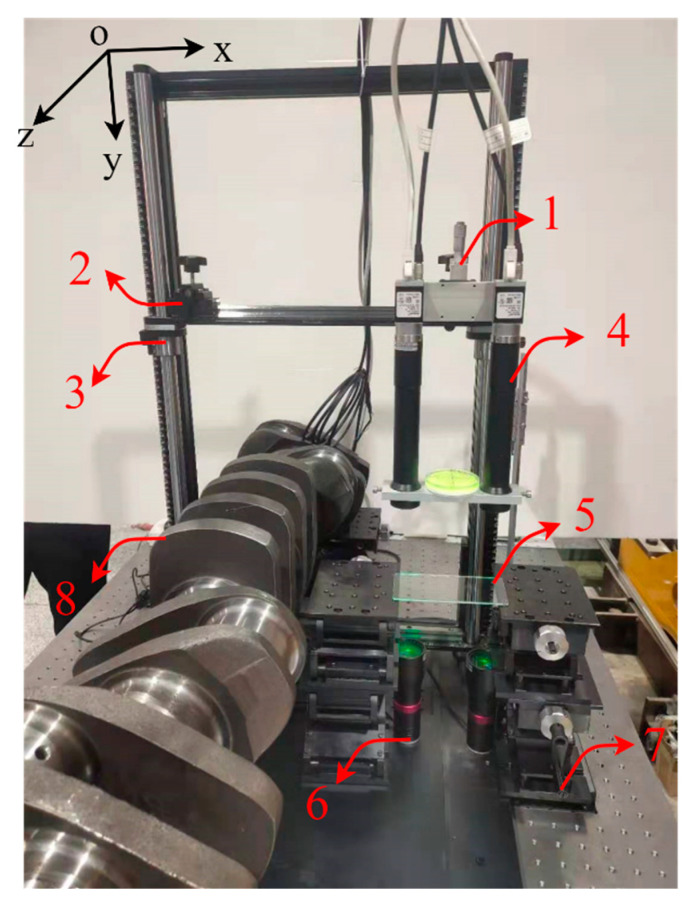
Measurement platform: (1) Adjustable device 1; (2) adjustable device 2; (3) adjustable device 3; (4) dual camera system; (5) calibration board; (6) parallel light sources; (7) adjustable device 4; (8) crankshaft.

The experimental measurement platform built in this paper is shown in Figure 8. The adjustment devices 1 and 3 can complete the fine adjustment along the *y*-axis direction and device 2 can complete the adjustment along the *x*-axis direction. Device 5 is a customized calibration board, device 6 comprises two parallel light sources, device 7 can complete the adjustment along the *y*-axis direction, and device 8 is the crankshaft.

### 3.3. Results of Calibration

**Figure 9:**
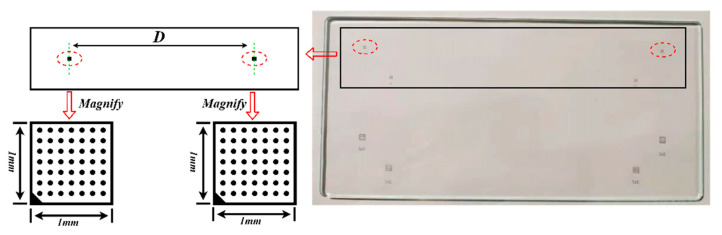
Calibration board.

According to the nominal diameter of the shaft to be measured, the specification of the calibration plate is determined by considering the field of view and measurement accuracy requirement. The calibration board used in the paper is shown in Figure 9. Two 7 × 7 circular patterns are printed on the calibration board, and the center distance is the nominal diameter D of the measured shaft. In this paper, the nominal diameter of the crankshaft diameter is 100 mm, and the pattern in the calibration plate adopts laser engraving technology, which ensure that D is 100 + 0.001 mm.

The inherent parameters of the cameras and lenses are listed in Table 1. The tolerance zone of crankshaft diameter is 0.012 mm and the field of view of the camera is 3.288 mm, so the center distance between the two cameras is adjusted to 100 ± 5 mm. We adjusted the height of the calibration plane to obtain a clear image in the calibration plate by the fine adjustment device 1. The depth of field of the lens is 0.72 mm.

The intrinsic and extrinsic parameters of the two cameras calculated by Zhang’s method [29] using 16 images of the calibration board are shown in Table 2 and Table 3.

As shown in Table 2 and Table 3, one camera has pillow distortion and the other has barrel distortion. However, in the follow-up experiments, the effect of camera distortion can be ignored, which fully proves the reliability of the telecentric camera in precision measurement.

### 3.4. Length Measurement of Calibration Board

**Figure 10:**
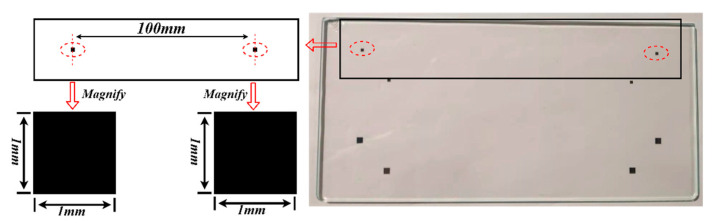
Schematic diagram of customized calibration board.

In order to test the stability and validity of the proposed method, the calibration board of known size, as shown in Figure 10, is measured. The theoretical center distance between the two black squares is 100.000 mm and the actual deviation is ±0.001 mm.

**Figure 11:**
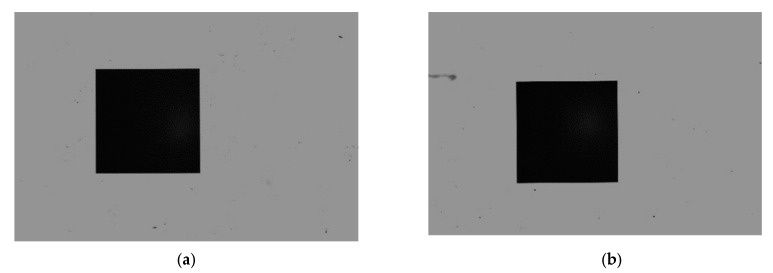
Images captured by the left and right camera. (**a**) The image captured by the left camera. (**b**) The image captured by the right camera.

The pictures captured by the left and right cameras are shown in Figure 11.

**Figure 12:**
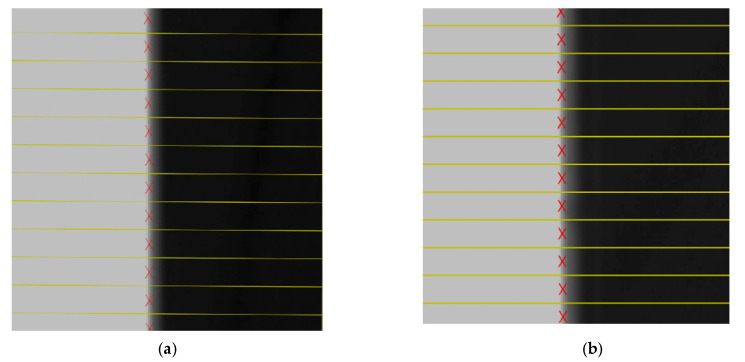
The subpixel coordinates of the images captured by camera. (**a**) The image captured by the left camera. (**b**) The image captured by the right camera.

**Figure 13:**
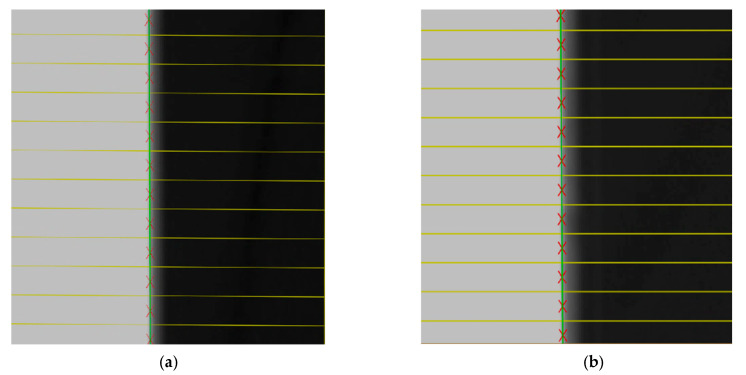
The fitting line of subpixel coordinates of the images captured by camera. (**a**) The image captured by the left camera. (**b**) The image captured by the right camera.

Subpixel detection described in Section 2.3 was applied to the images of the black squares, and the subpixel coordinates of the edges of the black square were calculated, as shown in Figure 12. The subpixel coordinates of the edges are fitted to the line equations by the least square method [30], as shown in Figure 13.

**Figure 14:**
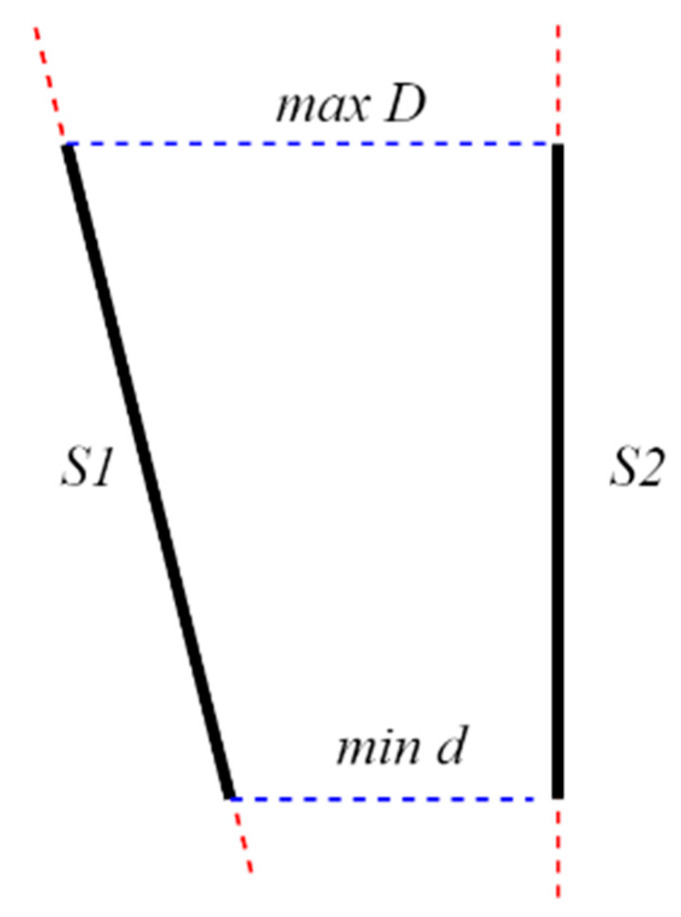
The distance calculation principle.

As shown in Figure 14, S1 and S2 are the line segments of left and right fitted image edges, respectively. In real applications, because S1 and S2 cannot be guaranteed to be completely parallel, the average of the maximum and minimum distances from the left fitted line segment to the right fitted line segment is used as the final distance.

The calibration board is moved at different positions within the field of view of the camera, and we collected 10 sets of images and measured the distance by the method proposed in this paper. The results are shown in the Table 4.

From the above table, it is obtained that these measurement results validate the effectiveness of the proposed method, which meets the measurement accuracy requirements of the calibration board with an accuracy requirement of 1 μm.

### 3.5. Diameter Measurement of Crankshaft

#### 3.5.1. Diameter Measurement of Crankshaft by the Proposed Method

**Figure 15:**
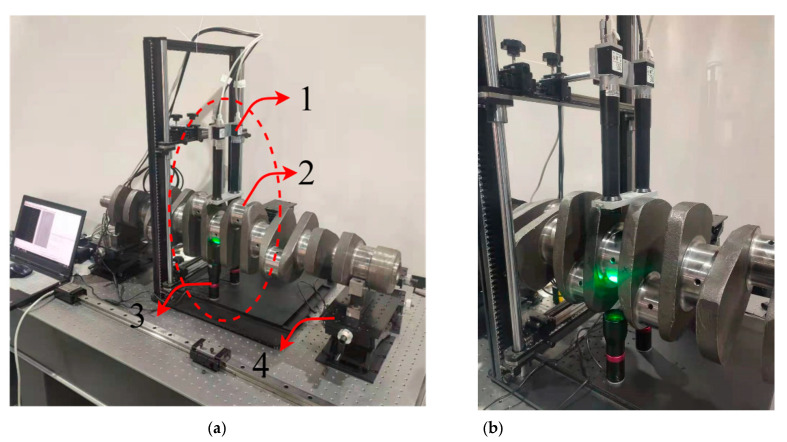
Measurement platform: (1) Dual camera system; (2) crankshaft; (3) parallel light sources; (4) adjustable V-lock. (**a**) Overall display image of platform. (**b**) Partial display image of platform.

As shown in Figure 15, the dual camera system is fixed above the crankshaft and the parallel light source is fixed under the crankshaft. We collected 10 sets of images at 36° intervals by rotating the crankshaft, and a total of 100 images were captured.

#### 3.5.2. Diameter Measurement of Crankshaft with CMM

**Figure 16:**
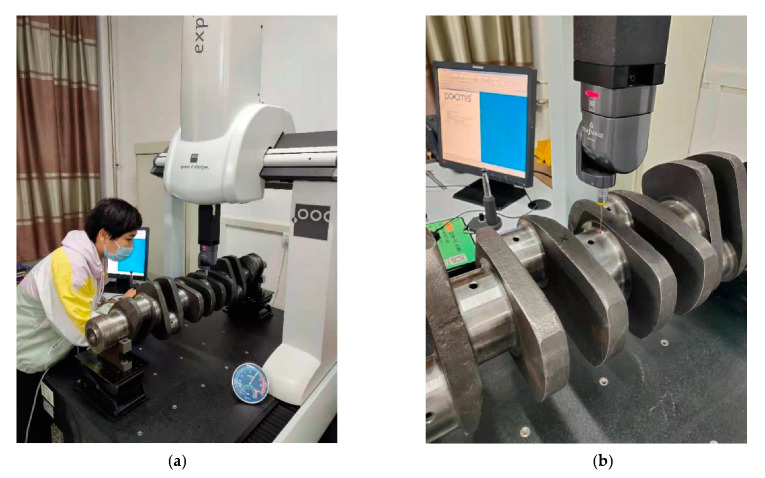
CMM platform. (**a**) Overall display image of CMM. (**b**) Partial display image of CMM.

The CMM was produced by Hexagon Metrology AB and the ambient temperature was 20 °C. ${\mathrm{MPE}}_{E}:\left(2.6+3.5\;L/1000\right)\;\mathrm{um}$,${\;\mathrm{MPE}}_{P}:2.2\;\mathrm{um}$. The crankshaft was fixed when the crankshaft diameter was measured by CMM. The centerline of the crankshaft was fitted by evenly selecting 20 points on the surface of the crankshaft diameter, and then the vertical plan S of the crankshaft centerline was calculated. The CMM measurement platform is shown in Figure 16.

#### 3.5.3. Experiment Results

Extensive experiments were carried out to verify the performance of the proposed method in comparison to CMM. The measurement results obtained by CMM and the proposed method are shown in Table 5.

Based on the above table, the proposed method is more stable compared with the CMM method, and both methods can be controlled with accuracy to the thousandth. The mean results of diameter measurements by different methods 10 times are shown in Table 6. $\bar{x}$ is the mean of the 10 measurements, and σ is the standard deviation. $\mu_{A}$ is the type A uncertainty, and $\mu_{95}$ is the expanded uncertainty when the confidence level is 95%.

From the above table, we observe that (1) the maximum $\mu_{95}$ values of CMM and the proposed method are ±2.11 μm and ±1.82 μm, and the half-interval length of the proposed method is shorter than CMM, and (2) the results indicate that the accuracy of the proposed method is equal to that of the CMM method.

#### 3.5.4. Measurement Error Analysis

**Figure 17:**
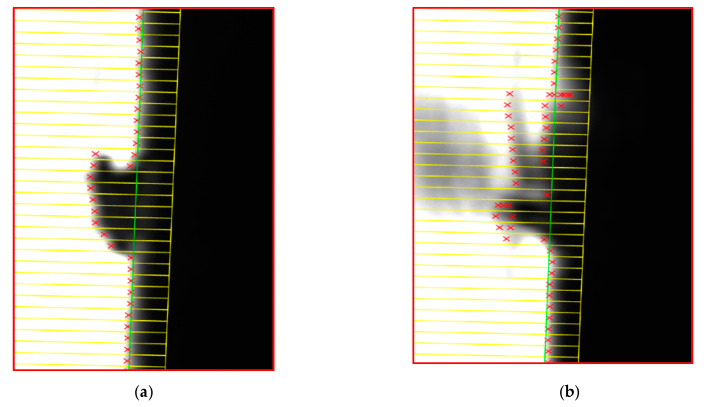
Influence of dust on image. (**a**) Influence of big dust on image. (**b**) Influence of complexed dust on image.

**Figure 18:**
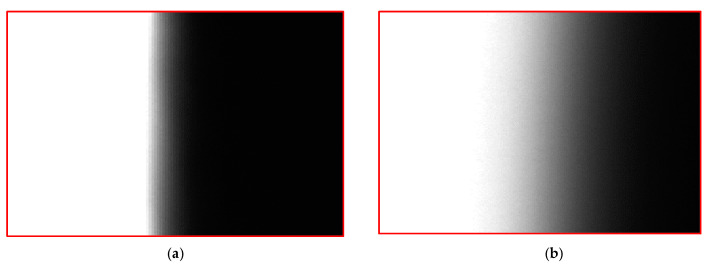
Influence of boundary. (**a**) Clear boundary. (**b**) Fuzzy boundary.

(1) As shown in Figure 17, the fitting accuracy of the edge will be affected by the electrostatic adsorption dust on the measured surface. To some extent, the measurement environment should be clean.

(2) Since the depth of field of the lens is 0.72 mm, if the position of the measurement system is not suitable, the boundary of the image will be blurred, which will reduce the fitting accuracy. The clear and blurred images are shown in Figure 18.

## 4. Conclusions

(1) We proposed here a method of high-precision measurement of a large-sized shaft based on a dual telecentric camera system to solve the problem that the measurement accuracy and the measurement range cannot be balanced in the shaft diameter measurement with machine vision. The calibration method of the two telecentric cameras was proposed based on Zhang’s calibration method, and we obtained the distance calculation formula by analyzing the imaging model of the proposed dual camera system.
(2) A customized high-precision calibration board was used to further improve the calibration accuracy of the dual camera system, and a subpixel detection algorithm based on quadratic interpolation and a line fitting algorithm were used to improve the measurement accuracy of the measured object.
(3) The measurement experiments of the calibration plate with a known center distance of 100.000 mm initially verified the effectiveness of the proposed method. The actual measurement results meet the measurement accuracy requirements of the calibration board.

In the same external environment, CMM and the proposed methods were used to measure a crankshaft diameter with a nominal diameter of 100. The maximum $\mu_{95}$ values of CMM and the proposed method were ±2.11 μm and ±1.82 μm, respectively, and the mean $\mu_{95}$ values of CMM and the proposed method were ±1.02 μm and ±1.07 μm, respectively, indicating that the measurement accuracy of the proposed method is roughly equal to that of the CMM.

(4) The factors affecting the measurement accuracy in the proposed method were analyzed, providing a basis for further research and the application of this method.

## Figures and Tables

**Table 1:** Inherent parameters of the cameras and lenses.

Index	Parameter Names	Parameter Values
1	Pixels of camera	5480 × 3648
2	Physical size of the camera chip	2.4 μm × 2.4 μm
3	Area of the camera chip	5480 × 2.4 = 13.152 mm 3648 × 2.4 = 8.7552 mm
4	Lens ratio	4
5	Field of the view	13.152/4 = 3.288 mm 8.7552/4 = 2.188 mm

**Table 2:** Calibrated parameters of the left camera.

Interior Parameters		Exterior Parameters	
Single pixel width (Sx)	2.41152 μm	X-coordinates	−0.241949 (mm)
Single pixel heigh (Sy)	2.4 μm	Y-coordinates	−0.156739 (mm)
Amplification factor	3.97793 (1/m^2^)	Z-coordinates	0 (mm)
Kappa (k)	−0.196915	Rotation in X direction	359.398°
Center point x-coordinates	2736.02 (pixel)	Rotation in Y direction	359.565°
Center point y-coordinates	1824.16 (pixel)	Rotation in Z direction	179.59°

**Table 3:** Calibrated parameters of the right camera.

Interior Parameters		Exterior Parameters	
Single pixel width (Sx)	2.414669 μm	X-coordinates	−0.179289 (mm)
Single pixel heigh (Sy)	2.4 μm	Y-coordinates	−0.579357 (mm)
Amplification factor	4.001188 (1/m^2^)	Z-coordinates	0 (mm)
Kappa (k)	9.64395	Rotation in X direction	6.189°
Center point x-coordinates	2481.53 (pixel)	Rotation in Y direction	0.325°
Center point y-coordinates	2610.27 (pixel)	Rotation in Z direction	179.788°

**Table 4:** The results of length measurements of 10 images.

Index	Min.	Max.	Mean
1	100.001	100.002	100.0015
2	100.000	100.001	100.0005
3	100.001	100.001	100.001
4	100.001	100.002	100.0015
5	100.000	100.001	100.0005
6	100.001	100.001	100.001
7	100.000	100.001	100.0005
8	100.001	100.001	100.001
9	100.001	100.001	100.001
10	100.001	100.002	100.0015

**Table 5:** The results of diameter measurements with a certain section of crankshaft (mm).

Index	CMM	The Proposed Method
1	99.9633	99.9663
2	99.9615	99.9663
3	99.9635	99.9664
4	99.9604	99.9662
5	99.9636	99.9662
6	99.9635	99.9663
7	99.9632	99.9663
8	99.9634	99.9664
9	99.9632	99.9663
10	99.9628	99.9663

**Table 6:** The mean results of diameter measurements 10 times.

Index	Measuring Results of CMM (mm)				Our Measuring Results (mm)			
	$\mathrm{Mean}\;\bar{x}$	Std. σ	$\mu_{A}$	$\mu_{95}$	$\mathrm{Mean}\;\bar{x}$	Std. σ	$\mu_{A}$	$\mu_{95}$
1	99.9628	0.00112	0.00035	±0.00069	99.9682	0.00240	0.00076	±0.00148
2	99.9638	0.00341	0.00108	**±0.00211**	99.9620	0.00173	0.00055	±0.00108
3	99.9645	0.00112	0.00035	±0.00069	99.9646	0.00165	0.00052	±0.00102
4	99.9639	0.00098	0.00031	±0.00061	99.9625	0.00294	0.00093	**±0.00182**
5	99.9638	0.00144	0.00045	±0.00089	99.9641	0.00147	0.00047	±0.00091
6	99.9640	0.00207	0.00065	±0.00128	99.9648	0.00148	0.00047	±0.00091
7	99.9630	0.00102	0.00032	±0.00063	99.9634	0.00198	0.00063	±0.00123
8	99.9636	0.00212	0.00067	±0.00131	99.9646	0.00134	0.00042	±0.00083
9	99.9639	0.00155	0.00049	±0.00096	99.9626	0.00130	0.00041	±0.00081
10	99.9632	0.00164	0.00052	±0.00102	99.9637	0.00266	0.00084	±0.00165
**Mean**	99.9636	0.00165	0.00055	±0.00102	99.9640	0.00190	0.00060	±0.00117

## Data Availability

The study did not report any data.
